# Gender-Specific Risk of Central Compartment Lymph Node Metastasis in Papillary Thyroid Carcinoma

**DOI:** 10.1155/2018/6710326

**Published:** 2018-03-11

**Authors:** Yushi Sun, Hongjun Lv, Shaoqiang Zhang, Yanxia Bai, Bingyin Shi

**Affiliations:** ^1^Department of Endocrinology, First Affiliated Hospital of Xi'an Jiaotong University, Xi'an, Shaanxi, China; ^2^Department of Otolaryngology-Head and Neck Surgery, First Affiliated Hospital of Xi'an Jiaotong University, Xi'an, Shaanxi, China

## Abstract

Our aim was to evaluate the impact of gender on the predictive factors of central compartment lymph node metastasis (CLNM) in papillary thyroid carcinoma (PTC). A retrospective study of 590 patients treated for PTC was performed. Univariate and multivariate analyses showed that gender (female; *P* = 0.001), age (≥45 y; *P* < 0.001), tumor size (>1 cm; *P* < 0.001), and multifocality (*P* = 0.004) were independent predictive factors of CLNM in PTC patients. Patients were divided into male group (*n* = 152) and female group (*n* = 438). Age (≥45 y; *P* = 0.001), T4 (*P* = 0.006) and multifocality (*P* = 0.024) were independent predictive risk factors of CLNM in male patients. As for female patients, age (≥45 y; *P* < 0.001), tumor size (>1 cm; *P* < 0.001), multifocality (*P* = 0.002), and microcalcification (*P* = 0.027) were independently correlated with CLNM. The sensitivity of the multivariate model for predicting CLNM in male patients was 64.9%, specificity was 82.9%, and area under the ROC curve (AUC) was 0.764. As for female patients, the sensitivity was 55.7%, specificity was 77.9%, and AUC was 0.73. This study showed that the predictive factors of CLNM indeed varied according to gender. To have a more accurate evaluation of CLNM, different predictive systems should be used for male and female patients.

## 1. Introduction

Papillary thyroid carcinoma (PTC), the most common type of thyroid cancer, constitutes 80% of all cases. The incidence of PTC increases rapidly and nearly doubles over the last 30 years [1]. PTC is usually considered as an indolent tumor and has a good prognosis. However, the cervical lymph node metastasis (LNM) especially central lymph node metastasis (CLNM) in PTC is very common with the incidence rate ranging from 45%–80% [2–5]. Several studies reported that LNM was associated with local tumor recurrence and poor prognosis of PTC [6–8]. However, there is substantial controversy regarding routine prophylactic central neck dissection in PTC patients because of the potential higher incidence of complications and uncertainty of improved oncological outcome [9, 10]. Accurate evaluation of LNM prior to surgery is important for determining the appropriate extent of lymph node dissection.

Neck ultrasound (US) is the most valuable method for evaluation of primary thyroid cancer and nodal status [11–13]. It has been demonstrated that US could detect 70%~93.8% LNM in the lateral neck. However, the sensitivity of US for predicting CLNM in PTC is only 23%~30.0% [11, 14]. Studies that attempted to investigate the predictive risk factors of CLNM in PTC patients were done recently and showed that some clinical factors such as age of onset, Hashimoto's thyroiditis (HT), thyroid-stimulating hormone (TSH), size of primary tumor, multifocality, and some other US features of the primary tumor were independently correlated with CLNM in PTC patients [3, 4, 15–17].

Gender is a prominent patient background parameter for PTC, and the clinicopathological features of PTC vary according to gender. It has been established that females have an earlier age of onset, but males tend to have a more aggressive disease with higher mortality [18–20]. Although previous studies investigated the factors predictive of CLNM in PTC, they did not distinguish between male and female patients. Our objective was to characterize and compare predictive factors of CLNM in PTC between the sexes. In this study, PTC patients were subclassified into male and female groups. The clinical features and their predictive value for CLNM were explored and compared between male and female patients.

## 2. Methods

A retrospective investigation was performed on PTC patients who had received initial thyroidectomy with at least one side central neck dissection at The First Affiliated Hospital of Xi'an Jiaotong University from January 2014 to June 2016. Central neck dissection was done routinely in our hospital for PTC patients in recent years. However, still, two percent of patients did not receive central neck dissection and were excluded from this study. Patients that had received preoperative I^131^ ablation or prior head and neck oncological surgery were excluded from this study. Those who had undergone TSH suppression therapy or antithyroid therapy before surgery were also excluded. Patients' age, gender, coexistence of HT (Hashimoto's thyroiditis), TSH (thyroid-stimulating hormone), T4 (tetraiodothyronine), T3 (triiodothyronine), TGAB (thyroglobulin antibody), TMAB (thyromicrosome antibody), ultrasonographic characteristics, and pathologic features were recorded.

A total of 590 patients were included in this study. The mean age was 42 years (range, 9–84 years). Coexistence of HT with PTC was confirmed by the postoperative pathological examination in 101 (17.12%) patients. CLNM was histologically proven in 58.64% (346/590) patients. Patients were divided into male group (*n* = 152) and female group (*n* = 438). The clinical features of PTC were compared between male and female groups. Univariate and multivariate analyses were performed to explore and compare the independent predictive risk factors of CLNM in male and female groups.

The measurements of preoperative serum thyroid function and thyroid relative autoantibodies were done by radioimmunoassay. The normal range for TSH, T4, and T3 was 0.25–5 *μ*IU/mL, 4.2–13.5 *μ*g/dL, and 0.8–2.2 ng/mL, respectively. The reference range of TGAB was <30% and TMAB was <20%.

The results of preoperative neck US examinations were reviewed. The ultrasonographic characteristics of the suspicious thyroid nodules, including size, numbers (multifocal/unifocal), location (bilateral/unilateral), shape (regular/irregular), border (clear/obscure), echogenicity (hypoechoic/hyperechoic or isoechoic), calcification (noncalcification/microcalcification/coarse calcification), and degree of vascularization (none/low/middle/high), were recorded. Thyroid nodules that were diagnosed TI-RADS fourth or fifth grade by radiologists were defined as suspicious thyroid nodules in this study. The diameter of the largest suspicious thyroid nodule was used as tumor size for analysis. Lymph node showing one or more suspicious features (focal or diffuse hyperechogenicity, presence of internal calcification, cystic change, round shape, or chaotic vascularity) on US was regarded as clinical pathologic lymph nodes. Complete datasets were not available for all patients due to differences in reported parameters.

The statistical analysis was performed with SPSS (version 22.0). *P* < 0.05 was considered statistically significant. Differences of single variables were tested with the chi-square test or unpaired nonparametric test (Mann–Whitney *U* test). Multivariate analysis using logistic regression analysis was performed on the variables that showed *P* < 0.1 in univariate analysis. Predictive value of those factors was measured using the area under the receiver operating characteristic (ROC) curve.

## 3. Results

### 3.1. Basic Clinical Features of PTC in Male Patients and Female Patients

9.27% (14/152) male PTC patients coexisted with HT which was significantly less than female patients (*P* = 0.003). Preoperative serum levels of TSH, TMAB, and TGAB were significantly lower in male patients than female patients (*P* < 0.05). A significant difference in nodular vascularization between male patients and female patients (*P* = 0.004) was noticed. There were no differences in age, preoperative T4 level, T3 level, nodular size, multifocality, bilaterality, nodular shape, border, internal echo, and calcification between male patients and female patients (Table 1).

### 3.2. Comparison of CLNM according to Gender

CLNM was histologically proven in 346 patients (58.64%). Approximately 71.05% (108/152) male patients and 54.33% (238/438) female patients exhibited CLNM (*P* < 0.001). Compared with female PTC patients, male PTC patients were significantly associated with the number of metastatic central lymph nodes (*P* < 0.001) but not the number of removed central lymph nodes (Table 1).

### 3.3. Predictive Risk Factors of CLNM in PTC Patients

Univariate and multivariate analyses were used to detect the risk factors of CLNM in PTC patients. It was showed that gender (female; *P* = 0.001), age (≥45 y; *P* < 0.001), tumor size (>1 cm; *P* < 0.001), and multifocality (*P* = 0.004) were independently correlated with CLNM in PTC patients (Tables 2 and 3).

### 3.4. Predictive Risk Factors of CLNM in Male Patients and Female Patients

Patients were divided into male group and female group. Univariate and multivariate analyses showed that the predictive factors of CLNM varied according to gender. In univariate analysis, age (<45 y/≥45 y) (*P* = 0.005), T4 (*P* = 0.026), and nodular margin (*P* = 0.012) were significantly associated with high prevalence of CLNM in male PTC patients. As for female patients, age (<45 y/≥45 y), tumor size (*P* < 0.001), multifocality (*P* = 0.05), and the presence of microcalcifications on US (*P* < 0.001) were significantly associated with CLNM (*P* < 0.001) (Table 4). Multivariate logistic regression analysis was performed to identify independent predictive risk factors of CLNM and showed that age (≥45 y) (OR 0.220; *P* = 0.001), T4 (OR 1.374; *P* = 0.006), and multifocality (multifocal) (OR 2.656; *P* = 0.024) were independent predictors of CLNM in male PTC patients (Table 5). As for female PTC patients, age (≥45 y) (OR 0.289; *P* < 0.001), tumor size (>1 cm) (OR 2.761; *P* < 0.001), multifocality (multifocal) (OR 1.966; *P* = 0.002), and microcalcification (OR 1.822; *P* = 0.027) were independently correlated with CLNM (Table 5).

### 3.5. ROC Analyses for Prediction of CLNM in PTC

**Figure 1:**
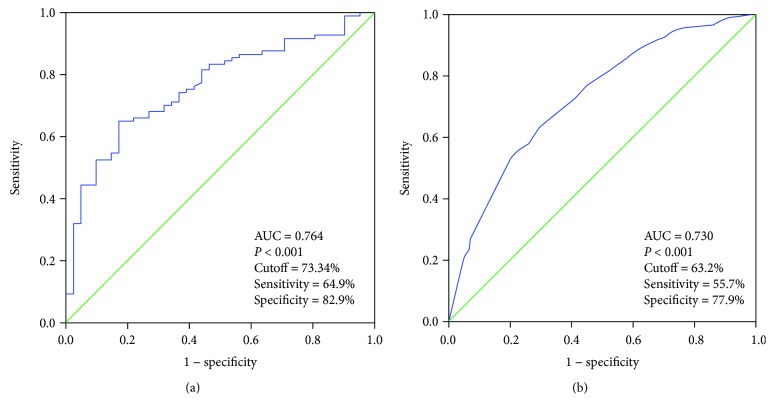
Receiver operating characteristic curve analyses for prediction of central lymph node metastases using the multivariate model. (a) Male papillary thyroid cancer patients. (b) Female papillary thyroid cancer patients.

Multivariate models were created and ROC analyses were performed to predict CLNM in male and female patients, respectively. The AUC was 76.4% for male and 73.0% for female patients (*P* < 0.001; Figures 1(a) and 1(b)). A cutoff point for prediction of CLNM was defined as a value 73.34% for male patients and 63.2% for female patients. The sensitivity of the multivariate model was 64.9%, and specificity was 82.9% for prediction CLNM in male PTC patients. For female patients, the sensitivity of the model was 55.7% and specificity was 77.9% (Figure 1).

## 4. Discussion

Lymph node metastasis especially central lymph node metastasis is very common in PTC with the incidence rate ranging from 45%–80% [2–5]. Accurate evaluation of CLNM prior to surgery is important for determining appropriate extent of lymph node dissection. A considerable body of literature shows that male gender, age of onset < 45 y, tumor size > 81.0 cm, and multifocality are associated with higher rate of CLNM in PTC [3, 4, 15–17]. Considering the impact of gender on the clinicopathological features of PTC, it is necessary to explore whether the predictive factors of CLNM differ by gender. This study confirmed the impact of gender on the clinical features of PTC and showed that the predictive factors of CLNM varied according to gender.

Gender is a prominent patient background parameter for PTC. Observed clinical and pathological differences between male and female PTC patients have been reported [18–20]. Previous studies showed that female PTC patients had an earlier age of onset, but men tend to have a more aggressive disease with higher mortality [21, 22]. In contradiction with previous reports, the current study showed no significant difference in age of onset, multifocality, and tumor size between male and female groups.

PTC has a strong propensity for lymph node metastasis. Nearly half of PTC has metastasized to the central lymph node at the time of diagnosis [2–5]. Preoperation risk of CLNM is important for deciding extent of surgery. Recent studies focused on the predictive risk factors for CLNM have revealed that age of onset, size of tumor, and multifocality are independently associated with CLNM in PTC [15, 17, 23]. It has been established that age is negatively correlated with CLNM in PTC, and the risk of CLNM increases proportionally with the number of tumor foci [15, 24]. Tumor size > 1 was also reported to be a risk factor of CLNM in PTC [25, 26]. Our study confirmed the predictive value of age and multifocality for CLNM in both male and female groups and showed that tumor size and microcalcification lost their predictive value for CLNM in male PTC patients.

TSH is a well-established risk factor of thyroid cancer. The association between TSH and CLNM was only observed by few of studies [3, 27]. The current study showed that although significant differences were observed in HT, TSH, TGAB, and TMAB between male and female groups, the predictive value of HT, TSH, TGAB, and TMAB for CLNM was not differed by gender. They were not independent risk factors of CLNM in the two groups. Instead, T4 was proved to be an independent predictive risk factor of CLNM in male patients.

An evaluation based on the predictive risk factors is more effective than the one based on US features of cervical lymph nodes to predict the CLNM in PTC patients. Different multivariate models were created to calculate the probability of CLNM for male and female patients, respectively. ROC analysis was performed to predict CLNM in both groups (AUCs: 76.4% and 73.0%, resp.). Compared with US of the cervical lymph node, the sensitivity of those models in predicting CLNM was much higher for male and female groups (64.9% versus 34.26% and 55.7% versus 32.35%, resp.), while the specificity of those models was a little lower than US (82.9% versus 88.64% and 77.9% versus 89.5%, resp.).

Several limitations of the present study should be noticed: First, this was a retrospective nonrandomized study. US was not performed by the same radiologist; interobserver variability among the radiologists may influence the results. Second, due to the difference in reported parameters by each radiologist, complete datasets were not available for all patients. Third, only PTC patients that received at least one side central neck dissection were included, and patients who had no LN dissection were excluded in this study, which has the potential selected bias associated with all retrospective analyses. Fourth, the number of patients especially the male PTC patients was relatively small. Further study with a large sample of patients is needed.

In conclusion, our study explored and confirmed the impact of gender on the predictive risk factors of CLNM in PTC for the first time. Our result suggested that to have a more accurate evaluation of CLNM, different predictive systems should be used for male and female patients.

## Figures and Tables

**Table 1:** Clinicopathologic characteristic of female and male PTC patients.

	Male (*n* = 152)	Female (*n* = 438)	*P* value
Age at diagnosis, M (range)	41 (9–78)	43 (12–84)	0.265
Age (<45 y/≥45 y)	92/60 60.5%/39.5%	244/194 55.7%/44.3%	0.301
Hashimoto's thyroiditis, *n* (%)	14 (9.27%)	87 (19.86%)	0.003
TSH, *μ*IU/mL, M (range)	1.63 (0.07–8.27)	2.02 (0.07–72.8)	0.017
T4, *μ*g/dL, M (range)	7.97 (4.21–13.2)	7.95 (2–25.9)	0.642
T3, ng/mL, M (range)	1.43 (0.52–2.55)	1.33 (0.5–4.57)	0.098
TGAB, %, M (range)	3.76 (2.45–53.6)	4.55 (1.82–63.7)	<0.001
TMAB, %, M (range)	3.08 (1.69–39.8)	3.54 (1.43–44)	0.001
*Ultrasonographic characteristics of suspicious nodules*			
Tumor size (≦1 cm/>1 cm)	35/117 23%/77%	104/334 23.7%/76.3%	0.488
Multifocal/unifocal	64/88 42.1%/57.9%	195/243 44.5%/55.5%	0.754
Bilateral/unilateral	42/110 27.6%/72.4%	134/304 30.6%/69.4%	0.515
Margin (regular/irregular)	60/84 41.7%/58.3%	179/228 44%/56%	0.721
Border (clear/obscure)	75/71 51.4%/48.6%	214/207 50.8%/49.2%	0.911
Hypoechoic/Hyper- or isoechoic calcification	139/10 93.3%/6.7%	418/20 95.4%/4.6%	0.313
Non-/micro-/coarse calcification	27/105/20 17.8%/69.1%/13.1%	93/277/66 21.3%/63.5%/15.1%	0.483
Vascularization None/low/middle/high	1/42/37/33 0.8%/37.2%/32.7%/29.2%	25/132/118/55 7.6%/40%/35.8%/16.7%	0.004
*Central lymph node metastasis*			
CLNM, *n* (%)	108 (71.05%)	238 (54.33%)	<0.001
Number of removed CLNs, M (range)	5 (0–27)	6 (0–41)	0.139
Number of metastatic CLNS, M (range)	2 (0–20)	1 (0–19)	<0.001

US: ultrasonography; CLNM: central lymph node metastases; NCLNM: central lymph node metastases negative; PTC: papillary thyroid cancer.

**Table 2:** Univariate analysis of the correlation between clinical factors of the primary tumor and rate of CLNM in PTC patients.

	CLNM (*n* = 346)	NCLNM (*n* = 244)	*P*
Gender (male/female)	108/238 31.2%/68.8%	44/200 18%/82%	<0.001
Age at diagnosis, M (range)	38 (9–79)	48 (13–84)	<0.001
Age (<45 y/≥45 y)	239/107 69.1%/30.9%	97/147 39.8%/60.2%	<0.001
Hashimoto's thyroiditis, present/absent	53/293 15.3%/84.7%	48/196 19.7%/80.3%	0.172
TSH, *μ*IU/mL, M (range)	1.99 (0.07–26.5)	1.79 (0.07–28.3)	0.676
T4, *μ*g/dL, M (range)	7.97 (4.36–24.4)	7.92 (3.3–15.1)	0.293
T3, ng/mL, M (range)	1.35 (0.5–2.55)	1.35 (0.63–2.28)	0.415
TGAB, %, M (range)	4.3 (1.98–62.6)	4.27 (1.82–63.7)	0.671
TMAB, %, M (range)	3.48 (1.53–41.7)	3.32 (1.69–44)	0.489
*Ultrasonographic characteristics of suspicious nodules*			
Tumor size (≦1 cm/>1 cm)	60/286 17.3%/82.7%	79/165 32.4%/67.6%	<0.001
Multifocal/unifocal	167/179 48.3%/51.7%	92/152 37.7%/62.3%	0.005
Bilateral/unilateral	110/236 31.8%/68.2%	66/178 27%/73%	0.235
Margin (regular/irregular)	129/194 39.9%/60.1%	110/118 48.2%/51.8%	0.042
Border (clear/obscure)	163/168 49.2%/50.8%	126/108 53.8%/46.2%	0.330
Hypoechoic/Hyper or isoechoic calcification	325/19 94.5%/5.5%	232/11 95.5%/4.5%	0.670
Non-/micro-/coarse calcification	53/243/48 15.4%/70.6%/14%	66/140/38 27%/57.4%/15.6%	0.001
Vascularization None/low/middle/high	12/99/87/59 4.7%/38.5%/33.8%/23%	14/75/68/29 7.5%/40.3%/36.6%/15.6%	0.661

CLNM: central lymph node metastases; NCLNM: central lymph node metastases negative; PTC: papillary thyroid cancer.

**Table 3:** Multivariate analysis of the correlation between clinical factors of the primary tumor and rate of CLNM in all PTC patients.

Variables	OR	CI	*P*
Gender (female)	0.475	0.304–0.744	0.001
Age (≥45 y)	0.264	0.181–0.386	<0.001
Margin (irregular)	1.378	0.947–2.006	0.094
Tumor size (>1 cm)	2.51	1.61–3.915	<0.001
Multifocality (multifocal)	1.766	1.202–2.595	0.004

CLNM: central lymph node metastases.

**Table 4:** Univariate analysis of the correlation between clinical factors of the primary tumor and rate of CLNM in male and female PTC patients.

	CLNM (*n* = 108)	Male	*P*	CLNM (*n* = 238)	Female	*P*
		NCLNM (*n* = 44)			NCLNM (*n* = 200)	
Age at diagnosis, M (range)	37 (9–78)	47 (26–66)	<0.001	38 (12–79)	47 (13–84)	<0.001
Age (<45 y/≥45 y)	73/35 67.6%/32.4%	19/25 43.2%/56.8%	0.005	166/72 69.7%/30.3%	78/122 39%/61%	<0.001
Hashimoto's thyroiditis, present/absent	10/98 9.2%/90.8%	4/40 9.1%/90.9%	1.0	43/195 18.1%/81.9%	44/156 22%/78%	0.304
TSH, *μ*IU/mL, M (range)	1.75 (0.07–7.27)	1.45 (0.07–5.79)	0.589	2.12 (0.07–26.5)	1.96 (0.07–28.3)	0.586
T4, *μ*g/dL, M (range)	8.34 (4.68–13.2)	7.86 (5.25–12)	0.026	8.03 (4.36–24.4)	8.17 (3.3–15.1)	0.892
T3, ng/mL, M (range)	1.39 (0.82–2.55)	1.42 (0.8–1.94)	0.149	1.34 (0.5–2.48)	1.33 (0.63–2.28)	0.823
TGAB, %, M (range)	3.84 (2.53–51.3)	3.51 (2.45–43.1)	0.423	4.79 (1.98–62.6)	4.39 (1.82–63.7)	0.408
TMAB, %, M (Range)	2.96 (1.9–39.8)	2.98 (1.69–29.7)	0.654	3.61 (1.53–41.7)	3.32 (2.01–44)	0.237
*Ultrasonographic characteristics of suspicious nodules*						
Tumor size (≦1 cm/>1 cm)	22/86 20.4%/79.6%	13/31 29.5%/70.5%	0.328	38/200 16%/84%	66/134 33%/67%	<0.001
Multifocal/unifocal	50/58 46.3%/53.7%	14/30 31.8%/68.2%	0.10	117/121 49.1%/50.9%	78/122 39%/61%	0.05
Bilateral/unilateral	32/76 29.6%/70.4%	10/34 22.7%/77.3%	0.388	78/160 32.8%/67.2%	56/144 28%/72%	0.280
Margin (regular/irregular)	36/65 35.6%/64.4%	24/19 55.8%/44.2%	0.012	93/129 41.9%/58.1%	86/99 46.5%/53.5%	0.352
Border (clear/obscure)	51/52 49.5%/50.5%	24/17 58.5%/41.5%	0.488	112/116 49.1%/50.9%	102/91 52.8%/47.2%	0.446
Hypoechoic/Hyper or isoechoic calcification	98/8 90.7%/9.3%	41/2 95.3%/4.7%	0.724	227/11 95.4%/4.6%	191/9 95.5%/4.5%	0.951
Non-/micro-/coarse calcification	18/73/17 16.7%/67.6%/15.7%	9/32/3 20.4%/72.7%/6.9%	0.322	35/170/31 14.8%/72%/13.2%	57/108/35 28.5%/54%/17.5%	<0.001
Vascularization None/low/middle/high	0/28/26/25 0/35.4%/32.9%/31.7%	1/14/11/8 2.9%/41.2%/32.4%/23.5%	0.434	12/71/61/34 6.7%/39.9%/34.3%/19.1%	13/61/57/21 8.6%/40.1%/37.5%/13.8%	0.579

CLNM: central lymph node metastases; NCLNM: central lymph node metastases negative; PTC: papillary thyroid cancer.

**Table 5:** Multivariate analysis of the correlation between clinical factors of the primary tumor and rate of CLNM in male and female patients.

Variables	OR	CI	*P*
*Male group*			
Age (≥45 y)	0.220	0.093–0.519	0.001
T4, *μ*g/dL	1.374	1.096–1.723	0.006
Multifocality (multifocal)	2.656	1.135–6.215	0.024
Margin (irregular)	1.241	0.549–2.807	0.604
*Female group*			
Age (≥45 y)	0.289	0.187–0.446	<0.001
Tumor size (>1 cm)	2.761	1.681–4.535	<0.001
Multifocality (multifocal)	1.966	1.284–3.010	0.002
*Calcification*			
Micro-/noncalcification	1.822	1.069–3.103	0.027
Coarse/noncalcification	1.427	0.712–2.858	0.316

CLNM: central lymph node metastases.
